# *SPL13* controls tomato lateral branch outgrowth by regulating brassinosteroid biosynthesis and signal transduction

**DOI:** 10.1093/hr/uhag007

**Published:** 2026-04-10

**Authors:** Long Cui, Fangyan Zheng, Lesong Jia, Feng Hu, Guo Ai, Jie Ye, Taotao Wang, Zhengming Wang, Zonglie Hong, Robert M Larkin, Zhibiao Ye, Junhong Zhang

**Affiliations:** Ganzhou Key Laboratory of Greenhouse Vegetables, College of Life Sciences, Gannan Normal University, Ganzhou, Jiangxi 341000, China; National Key Laboratory for Germplasm Innovation & Utilization of Horticultural Crops, Huazhong Agricultural University, Wuhan 430070, China; Ganzhou Key Laboratory of Greenhouse Vegetables, College of Life Sciences, Gannan Normal University, Ganzhou, Jiangxi 341000, China; National Key Laboratory for Germplasm Innovation & Utilization of Horticultural Crops, Huazhong Agricultural University, Wuhan 430070, China; Ganzhou Key Laboratory of Greenhouse Vegetables, College of Life Sciences, Gannan Normal University, Ganzhou, Jiangxi 341000, China; Ganzhou Key Laboratory of Greenhouse Vegetables, College of Life Sciences, Gannan Normal University, Ganzhou, Jiangxi 341000, China; National Key Laboratory for Germplasm Innovation & Utilization of Horticultural Crops, Huazhong Agricultural University, Wuhan 430070, China; National Key Laboratory for Germplasm Innovation & Utilization of Horticultural Crops, Huazhong Agricultural University, Wuhan 430070, China; National Key Laboratory for Germplasm Innovation & Utilization of Horticultural Crops, Huazhong Agricultural University, Wuhan 430070, China; National Key Laboratory for Germplasm Innovation & Utilization of Horticultural Crops, Huazhong Agricultural University, Wuhan 430070, China; Department of Plant Sciences, University of Idaho, Moscow, ID 83844, USA; National Key Laboratory for Germplasm Innovation & Utilization of Horticultural Crops, Huazhong Agricultural University, Wuhan 430070, China; National Key Laboratory for Germplasm Innovation & Utilization of Horticultural Crops, Huazhong Agricultural University, Wuhan 430070, China; National Key Laboratory for Germplasm Innovation & Utilization of Horticultural Crops, Huazhong Agricultural University, Wuhan 430070, China

## Abstract

Plant architecture can directly impact on tomato (*Solanum lycopersicum*) fruit production. Our previous work has demonstrated an important role of microRNA156a (miR156a) in determining the lateral branches mainly by regulating *SQUAMOSA PROMOTER BINDING PROTEIN LIKE 13* (*SPL13*) expression in tomato. However, the regulatory pathway by which the miR156a-SPL13 module regulates branching remains obscure. In this study, we examined the relationship between *SPL13* and two other genes, *BRANCHED1b* (*BRC1b*) and *DWARF* (*DWF*), which have previously been known to regulate lateral branch development in tomato. Our findings indicate that SPL13 directly interacts with the promoters of *BRC1b* and *DWF*, enhancing *BRC1b* expression while inhibiting *DWF* expression, thereby finely regulating lateral branch development in tomatoes. Additionally, overexpression of *BRC1b* or knockout of *DWF* were found to rescue the increased lateral branch outgrowth phenotype of the *spl13* mutant plants. Furthermore, brassinosteroid (BR) content was high in *spl13* mutant plants, supporting the notion that SPL13 negatively regulates BR biosynthesis. These findings suggest that SPL13 serves as an activator of the BR signaling downstream gene *BRC1b* and a repressor of the BR biosynthesis gene *DWF* to regulate lateral branch outgrowth in tomato.

One-sentence summary: The miR156a-targeted transcription factor SlSPL13 regulates plant architecture in tomato.

## Introduction

Tomato is a globally significant vegetable crop. It also serves as a model plant for research on floral and fruit development in perennial, sympodial plants. Plant architecture is a major determinant of crop yield. Artificial selection for improved plant architecture that has facilitated increases in flower production and yield has occurred throughout the domestication and improvement of tomato. Various regulators play a role in shaping optimal plant architecture, as well as influencing flower quantity, fruit size, and yield. Identifying and characterizing these regulatory genes is crucial for enhancing our understanding of the genetic foundations of phenotypic variation and aiding in the breeding of superior varieties.

MicroRNAs (miRNAs) affect plant development by cleavage or repression of specific mRNAs [1, 2]. miR156 modulates a broad gene network related to plant development by suppressing *SPL* gene family expression [3–7]. There are 17 SPL genes in the tomato genome, among which seven *SPLs* are targets of miR156a [1, 8]. Similarly, miR156 targets 11 *SPLs* in rice and 10 *SPLs* in Arabidopsis [9, 10]. *SPL* genes encode plant-specific transcription factors involved in regulating various plant growth processes. Grain yield is regulated by several miR156-targeted *SPLs*, including *SPL13*, *SPL14*, and *SPL16* in rice [11–15]. Rice *SPL14*, also referred to as *Ideal Plant Architecture1* (*IPA1*), influences yield and disease resistance [16]. In Arabidopsis, miR156 targets the *SPL3*, *SPL4*, *SPL5*, *SPL9*, and *SPL15* genes, which are known to influence shoot growth, flowering, growth cycle regulation, plastochron length, and organ size [17–25]. In tomatoes, miR156 targets the SPL gene, which regulates the *Colorless nonripening* (*Cnr*) gene responsible for fruit ripening [26]. *SlSPL13* regulates inflorescences, lateral branches, and fruit size, and is also a target of miR156 [1, 8].

Plant architecture determines light perception, photosynthesis, and crop productivity in plant. Higher plants architecture is established mainly through lateral meristems activity, which greatly affect most fundamental aspects of the plant life [27–29]. Recent research indicates that SPL transcription factors, along with proteins like DWARF53, Teosinte Branched 1 (TB1), and Barren Stalk 1 (BA1), play a role in regulating plant architecture in rice and bread wheat [30–33]. In bread wheat, miR156-targeted SPLs regulate the expression of *TB1* and *BA1*, influencing plant architecture [31]. In rice, the OsSPL14 influences plant architecture by binding to the promoter of *TB1* and consequently suppressing tillering [34]. Significantly, OsSPL14 binds to the *OsDWARF53* promoter and affects strigolactone (SL)-induced *OsDWARF53* expression by the feedback regulation [32]. In Arabidopsis, the transcription factor SPL13 regulates cell division orientation and thereby influences root growth [35]. In tomato, SlSPL13 regulates inflorescence morphogenesis by directly activating *SINGLE FLOWER TRUSS* (*SFT*) [8]. It also affects lateral branch development by directly regulating the cytokinin (CK) biosynthesis gene *ISOPENTENYL TRANSFERASES 1* (*IPT1*) and the SL biosynthesis genes *CAROTENOID CLEAVAGE DIOXYGENASE 7* (*CCD7*) and *MORE AXILLARY GROWTH 1* (*MAX1*) [36]. However, the mechanism by which the miR156a–*SlSPL13* module regulates lateral branch development in tomato requires further investigation.

Brassinosteroids (BRs) are essential steroid hormones that influence plant growth and development [37, 38]. *CYP724B* and *CYP90B*, which are tissue-specific BR biosynthesis genes, regulate tiller number in rice [39]. TB1 (TCP/Cycloidea/PCF) acts downstream of BR signaling, has been shown to affect the outgrowth of buds in different plant species [38, 40–43]. The gene BRANCHED1b (BRC1b), an ortholog of TB1 in eudicots, is specifically expressed in axillary buds and serves as a key regulator inhibiting bud outgrowth [29, 41]. Tomato plants of the *dwf* mutant exhibit deficiencies in BR biosynthesis and develop significantly fewer lateral branches than the control plants. In contrast, overexpressing *DWF* has been shown to affect lateral bud outgrowth in tomato [38]. Although the function of BRC1b, DWF, and SPL13 in lateral branch arrest is widely conserved in the plant kingdom, the direct molecular links between SPL13 and BRC1b and DWF in tomato remain unknown.

Our previous work has demonstrated that miR156a targets the *SPL13* gene and regulates lateral branch development in tomato [1, 8]. In this report, we discovered that both *BRC1b* and *DWF* acted as potential regulators of SlSPL13. Overexpression of *BRC1b* was found to revert the increased lateral branch outgrowth phenotype in the *spl13* knockout lines, which is consistent with the proposed function of *BRC1b* acting downstream of SPL13. The observation that the lateral branch defects of the *spl13/dwf* double mutant and *dwf* single mutant are phenotypically similar implies that *DWF* may have a critical function downstream of SPL13. Our findings also indicate that SPL13 may inhibit BR biosynthesis in tomato. These findings enhance our comprehension of the miR156a–*SlSPL13–DWF/BRC1b* module, which is crucial for shaping plant architecture in tomato.

## Results

### SPL13 regulates lateral branch development in tomato

**Figure 1:**
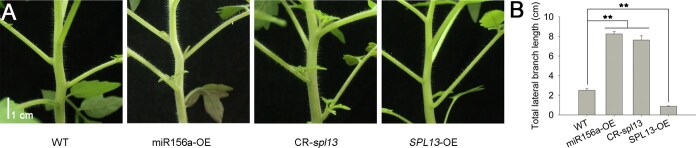
Lateral branch phenotypes of transgenic tomato plants overexpressing miR156a or *SPL13*, and *SPL13* knockout plants. (A) Images of lateral branch phenotypes from 5-week-old representative lines of miR156a-OE, CR-*spl13*, *SPL13*-OE, and WT/AC plants. CR, gene knockout lines created using CRISPR/Cas 9 technology. Scale bar, 1 cm. (B) Mean values with standard errors (±SE) of the total lateral branch length were recorded using three transgenic lines and WT. Twelve biological replicates from each transgenic line were independently analyzed for statistical comparison. *t*-tests were used to identify statistically significant differences between mean values, indicated by asterisks for ^**^P < 0.01.

In this work, we first examined phenotypes of lateral branch development in transgenic tomato lines overexpressing miR156a (miR156a-OE) and overexpressing *SPL13* (*SPL13*-OE/flag), and in CRISPR-mediated *SPL13*-gene knockout plants (CR-*spl13*). We observed that the growth of lateral branches was suppressed in the *SPL13*-OE/flag lines but enhanced in the miR156a-OE and CR-*spl13* lines (Fig. 1A). In 5-week-old seedlings, the lateral branches of miR156a-OE and CR-*spl13* lines were three times longer than those of wild-type (WT) plants (Fig. 1B). The SPL13-OE/flag lines exhibited lateral branches with a total length approximately one-third that of the WT (Fig. 1B). Taken together, these results reveal that *SPL13* is essential for the lateral branch outgrowth in tomato.

### SPL13 regulates the expression of *BRC1b* and *DWF*

**Figure 2:**
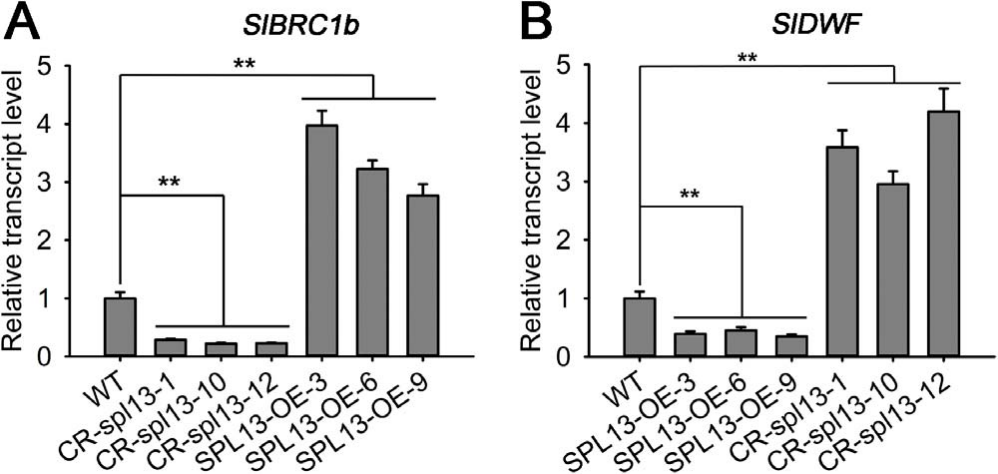
Relative transcript levels of *BRC1b* and *DWF* genes in shoot apices of *SPL13*-overexpressing lines and spl13 knockout mutant lines. Total RNA was extracted from shoot tips of 5-week-old seedlings. Relative transcript levels of *BRC1b* (A) and *DWF* (B) were quantified using RT-qPCR using total RNA from SPL13-flag overexpressing lines (SPL13–3, −6, −9) and CR-*spl13* mutant lines (−1, −10, −12). The transcript expressions of *BRC1b* and *DWF* in the control (WT) plants were set at 1.0. Mean values and ± SE were based on three replicates. Asterisks (^**^) indicate statistically significant differences relative to WT (*P* < 0.01; Student’s *t*-test).

Previous studies [29, 38] have identified the *BRC1b* and *DWF* genes as crucial regulators of lateral branch outgrowth in tomato. The length of lateral branches has been shown to increase significantly in the *DWF*-OE transgenic lines and *brc1b* mutant plants, while the growth of lateral branches is drastically suppressed in the *BRC1b*-OE and *dwf* mutant plants [29, 38]. Our results showed that the lateral branches of CR-*spl13* plants were phenotypically similar to the *brc1b* and *DWF*-OE plants and that the *SPL13*-OE lines phenotypically resembled the *BRC1b*-OE and *dwf* mutant plants (Fig. 1). Our previous work has also demonstrated that SPL13 functions as a transcription factor and its protein accumulates in the nucleus [8]. These data prompted us to raise the hypothesis that SPL13 may regulate lateral branch development by upregulating *BRC1b* and downregulating *DWF* expression. *BRC1b* expression was notably decreased in CR-*spl13* lines and increased in *SPL13*-OE lines compared to WT. In contrast, with *DWF*, we observed the opposite expression patterns (Fig. 2). Thus, the miR156a–SPL13 module appears to function as the core factor regulating the expression of BRC1b and DWF genes.

### SPL13 downregulates *DWF* but upregulates *BRC1b* gene expression

**Figure 3:**
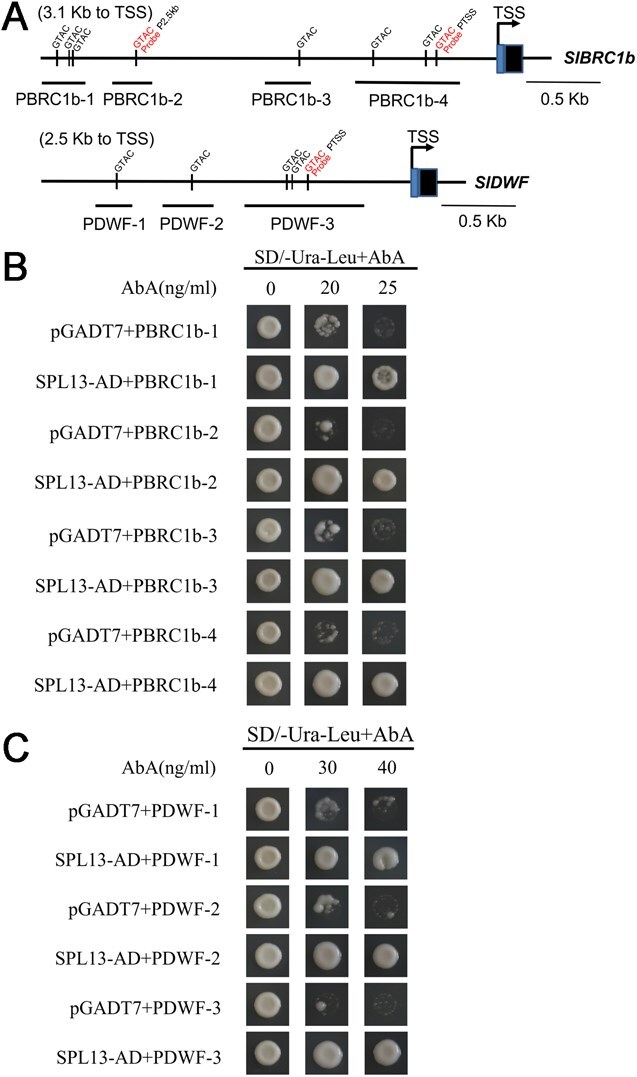
Y1H assays were conducted to assess SPL13 binding to the *BRC1b* and *DWF* promoters. (A) Diagram illustrating the 3095-bp *BRC1b* and 2136-bp *DWF* promoter regions. The promoter of *BRC1b* contains eight GTAC *cis*-elements, while *DWF* has five. In the Y1H assay, four constructs with distinct promoter fragments (PBRC1b-1, PBRC1b-2, PBRC1b-3, and PBRC1b-4) from *BRC1b* and three constructs with different promoter fragments (PDWF-1, PDWF-2, and PDWF-3) from *DWF* were utilized. PBRC1b-1, PBRC1b-2, PBRC1b-3, and PBRC1b-4 contain from −3095 to −2801 bp, −2624 to −2333 bp, −1581 to −1266 bp, and from −983 to −240 bp relative to the translational start site (TSS), respectively. PDWF-1, PDWF-2, and PDWF-3 contain from −2136 to −1909 bp, −1684 to −1360 bp, and from −1238 to −366 bp relative to the TSS, respectively. (B) Y1H analysis of SPL13 binding to the distinct core sequences within the *BRC1b* and *DWF* promoters. The bait vectors, PBRC1b-1, PBRC1b-2, PBRC1b-3, PBRC1b-4, PDWF-1, PDWF-2, and PDWF-3 and the SPL13 prey vector were transferred into Y1H gold. The enhanced resistance of yeast cells to antibiotic aureobasitin A (AbA) was employed to assess the interaction between the bait (promoter fragment) and prey (*SPL13*). Cotransformation of the bait vectors, PBRC1b-1, PBRC1b-2, PBRC1b-3, PBRC1b-4, PDWF-1, PDWF-2, and PDWF-3, with either pGADT-Rec2-53 or pGADT7 served as positive and negative controls, respectively.

SPLs regulate their target genes by binding to GTAC *cis*-elements within the gene promoters [22, 23]. In this work, we investigated the potential direct binding of SPL13 to the *BRC1b* and *DWF* promoters by initially examining these promoters for GTAC elements. We successfully found eight and five GTAC elements in the *BRC1b* and *DWF* promoter, respectively (Fig. 3A). To assess the significance of GTAC elements, four reporter constructs were designed for the *BRC1b* promoter: PBRC1b-1 (−3095 to −2801 bp), PBRC1b-2 (−2624 to −2333 bp), PBRC1b-3 (−1581 to −1266 bp), and PBRC1b-4 (−983 to −240 bp) relative to the translational start codon. Similarly, three reporter constructs were designed for the *DWF* promoter, as such PDWF-1 containing the fragment from −2136 to −1909 bp, PDWF-2 from −1684 to −1360 bp, and PDWF-3 from −1238 to −366 bp (Fig. 3A). These promoter–reporter constructs were tested in yeast one-hybrid (Y1H) assays. This result clearly showed that SPL13 could bind to all of the tested promoter fragments in the four *BRC1b* and three *DWF* constructs (Fig. 3B and C).

**Figure 4:**
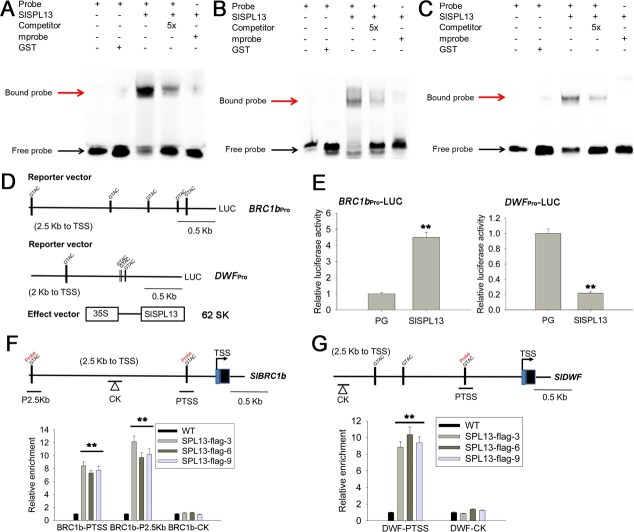
Binding of SPL13 to the promoter of *BRC1b* and *DWF* by EMSAs, dual-LUC assay, and ChIP-qPCR analysis. (A–C) EMSAs examining the binding of SPL13 to the promoters of *BRC1b* (A, B) and *DWF* (C). DNA probes were the genomic DNA fragments containing the core GTAC motif from each of the two gene promoters. Mutated probes, mprobe, were the same genomic DNA fragments in which the core binding element, GTAC, was replaced with AAGA. mprobe also served as a negative control. Competitors were the same genomic DNA fragments but were nonlabeled. Competitors were added at 5-fold molar excess (5×) relative to their corresponding biotin-labeled probes. The symbols ‘–’ and ‘+’ denote the absence and presence of the respective protein or probes. Upper arrows indicate the expected position of DNA–protein complex. Lower arrows denote unbound free probes. (D) Schematic diagrams of dual-LUC reporter plasmids and SlSPL13 effector plasmid. The dual-LUC reporters were under the control of the 2513-bp *BRC1b* promoter and 1565-bp *DWF*, respectively. The SPL13 effector was expressed under the control of the CAMV 35S promoter. (E) Effect of SlSPL13 on the activities of the *BRC1b* and *DWF* promoters. Plasmids for the dual-LUC assay were transiently expressed in *N. benthamiana* leaves via *A. tumefaciens*-mediated transformation. PG, the empty vector (pGreen II 62-SK). The level of LUC activity in the empty vector (PG) was set at 1.0, and the activation values in *BRC1b*_Pro_-LUC and *DWF*_Pro_-LUC were presented as fold of increase relative to the value of the empty vector (PG). Each value represents the mean from eight biological replicates. Asterisks indicate statistically significant differences that were determined using the *t*-test: ^**^P < 0.01. (F, G) ChIP-qPCR analysis of the binding of SPL13 to the *BRC1b* and *DWF* promoters in WT and three 35S-SPL13-Flag lines (−3, −6, and − 9). Schematic diagrams of the 2513-bp *BRC1b* (F) and 2500-bp *DWF* (G) promoter regions are shown (top). The promoter fragments enrichment in ChIP-qPCR analysis from young tissue of the transgenic plants was quantified using qPCR (bottom). The data were normalized to WT plants. The ChIP-qPCR analysis set the relative enrichment of promoter fragments in WT to a baseline value of 1. Mean values with ±SE were taken from three replicates. Asterisks (^**^) indicate statistically significant differences relative to WT (*P* < 0.01; Student’s *t*-test).

An electrophoretic mobility shift assay (EMSA) was conducted using a recombinant SPL13 glutathione S-transferase (GST) fusion protein to further investigate SPL13 binding to the GTAC elements of *BRC1b* and *DWF*. We found that SPL13, but not the control protein GST, could bind to DNA fragments containing the core *cis*-element, GTAC, which was consistent with the conclusion from Y1H assays (Fig. 4A–C). When the core binding element, GTAC, was mutated, the binding between SPL13 and the promoter DNA fragments disappeared. Based on the Y1H and EMSA results (Fig. 3 and Fig. 4A–C), we conclude that SPL13 binds to the GTAC element in the promoters of *BRC1b* and *DWF* genes.

To test whether the expression of *BRC1b* and *DWF* genes is directly regulated by SPL13, we performed dual-luciferase (LUC) assays by transiently coexpressing either 35S-SlSPL13 and *BRC1b*_Pro_-LUC or 35S-SlSPL13 and *DWF*_Pro_-LUC constructs in tobacco leaves (Fig. 4D). As a negative control, 35S-SlSPL13 was replaced with pGreen II 62-SK empty vector (PG). The LUC activity increased dramatically when 35S-SlSPL13 was coexpressed with *BRC1b*_Pro_-LUC (Fig. 4E, left) but decreased significantly when *DWF*_Pro_-LUC was coexpressed with 35S-SlSPL13 (Fig. 4E, right). Additionally, we performed chromatin immunoprecipitation followed by quantitative PCR (ChIP-qPCR) assays with the *BRC1b* and *DWF* promoters in the SPL13-flag lines to confirm the ChIP-seq results. The results showed that two *BRC1b* promoter fragments (P2.5Kb and PTSS) and one *DWF* promoter fragment (DWF-PTSS) that each contain a core GTAC sequence were highly enriched in the pull-down sequences of the SPL13-Flag line (Fig. 4F, G), suggesting that SPL13 protein binds to the core GTAC *cis-*element of these promoter fragments. The *BRC1b* and *DWF* promoter fragments without the GTAC sequence (CK) did not show enrichment by SPL13-flag (Fig. 4F, G). Our analysis indicates that SPL13 modulates *BRC1b* and *DWF* expression by interacting with their core GTAC sequences. These findings indicate that SPL13 influences tomato plant development by interacting with GTAC elements of *BRC1b* and *DWF* genes, thereby modulating their expression.

### Either *DWF* knockout or *BRC1b* overexpression rescues the lateral branching phenotype of *spl13*

**Figure 5:**
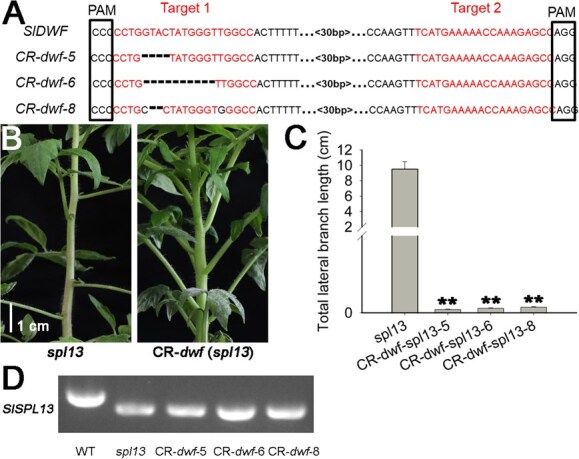
Branch phenotypes of CR-*dwf* lines in the *spl13* background. (A) Mutant alleles of *dwf*. Deletions of 4, 10, and 2 bp in *DWF* were detected in three transgenic lines (CR-*dwf*-5, -6, and -8) created using CRISPR/Cas9 technology, which aligned against that of the WT *DWF*. The target sequences of sgRNA are highlighted, while the protospacer-adjacent motif (PAM) sequences are enclosed in boxes. (B, C) Analysis of lateral branches from *spl13* and *dwf spl13* double mutants. Total lateral branch length was quantified in 35-day-old *spl13* and *dwf spl13* double mutants. Mean values with ±SE for the total lateral branch length are from three transgenic lines and *spl13*. Each transgenic line was subjected to independent analysis of twelve biological replicates to facilitate statistical comparison. Asterisks (^**^) denote statistically significant differences relative to *spl13* (*P* < 0.01; Student’s *t*-test). (D) PCR products from the *SPL13* gene were amplified in transgenic, *spl13*, and WT plants.

**Figure 6:**
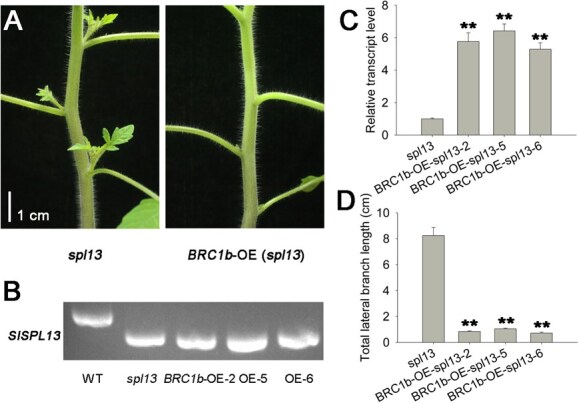
Branch phenotypes of *BRC1b*-OE/flag lines in the *spl13* background. (A) Lateral branch phenotype of *spl13* and *BRC1b*-OE lines in the *spl13* background. (B) PCR products amplified from the *SPL13* gene in transgenic, *spl13*, and WT plants. (C) Relative expression of *BRC1b* in shoot tips of 5-week-old *BRC1b*-OE *spl13* seedlings. The qRT-PCR data were normalized to expression in *spl13* plants, which was set to 1. Mean values with ±SE are based on three biological replicates. Asterisks (^**^) indicate statistically significant differences relative to *spl13* (*P* < 0.01; Student’s *t*-test). (D) Analysis of lateral branches from *spl13* and *BRC1b*-OE *spl13* lines. Total lateral branch length was quantified in 35-day-old *spl13* and in *BRC1b*-OE *spl13* lines. Mean values with ±SE for the total lateral branch length are from three transgenic lines and *spl13*. Twelve biological replicates from each transgenic line were independently analyzed for statistical comparisons. Asterisks (^**^) indicate statistically significant differences relative to *spl13* (*P* < 0.01; Student’s *t*-test).

To test whether SPL13 controlled lateral branch development by regulating *DWF* and *BRC1b*, we examined the genetic relationship between *SPL13* and either *DWF* or *BRC1b*. We generated *DWF* CRISPR/Cas9 knockout (CR-*dwf*) and *BRC1b* overexpression (OE/flag) lines in the *spl13* mutant background. The CR-*dwf* lines were generated using guide RNAs targeting an 84-bp region of the *DWF* gene (Fig. 5A). We detected base deletions in different CR-*dwf* lines (Fig. 5A). The *dwf spl13* double mutant plants exhibited a significantly reduced total length of lateral branches compared to *spl13* plants (Fig. 5B–D). These data revealed that DWF could alter the effect of SPL13 on the tomato lateral meristems outgrowth. The expression level of *BRC1b* was significantly higher in *BRC1b*-OE/flag lines 2, 5, and 6 than in the *spl13* mutant. The *BRC1b*-OE/flag *spl13* plants exhibited a significant reduction in the total length of lateral branches compared to *spl13* plants (Fig. 6). These findings align with the role of *DWF* and *BRC1b* as downstream regulators of SPL13 in controlling lateral branch outgrowth in tomato.

### SPL13 downregulates BR biosynthesis in tomato

**Figure 7:**
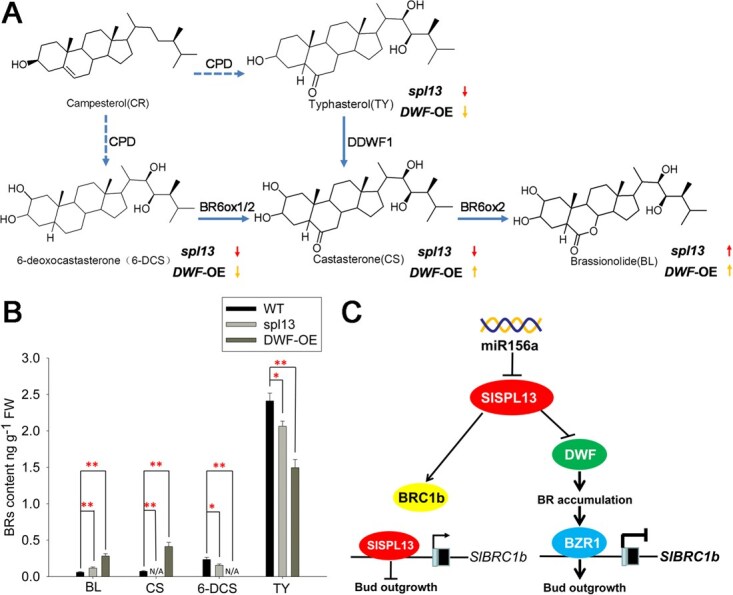
SPL13 regulates brassinosteroid biosynthesis and signaling. (A) Chemical structures of intermediates in BR biosynthesis. Upper arrows indicate changes in BR intermediate levels in spl13 plants. Lower arrows indicate changes in BR intermediate levels in DWF-OE plants. (B) Contents of BL, CS, 6-DCS, and TY. Mean values and ± SE were based on three biological replicates. Statistically significant differences were determined using the *t*-test and are indicated by asterisk (^*^) at *P* < 0.05 or (^**^) at *P* < 0.01. (C) Model of regulation of lateral branch outgrowth in tomato by the miR156a-SPL13 module. In this model, the transcript level of SPL13 is negatively controlled by miRNA156a. SPL13 enhances BRC1b expression to suppress bud outgrowth while simultaneously inhibiting DWF expression, thereby promoting lateral branch outgrowth by regulating BR synthesis and signaling. BZR1, BRASSINAZOLE-RESISTANT 1.

To investigate if SPL13 influences lateral branch outgrowth through BR synthesis in tomato, we quantified levels of BR derivatives brassinolide (BL), typhasterol (TY), castasterone (CS), and 6-deoxocastasterone (6-DCS) in *DWF*-OE transgenic and *spl13* mutant plants, comparing them to control (WT) plants. BL is the most active BR derivative, with CS as its immediate biosynthetic precursor, while TY and 6-DCS serve as precursors to CS (Fig. 7A). Our results showed that both *DWF*-OE and *spl13* plants produced higher levels of BL but accumulated less TY and 6-DCS than control (Fig. 7B). The levels of CS were lower in *spl13* but higher in *DWF*-OX than WT plants (Fig. 7B). Altogether, these results indicate that SPL13 downregulates BR biosynthesis to affect lateral branch outgrowth in tomato.

## Discussion

### The miR156a–*SPL13* module regulates plant architecture through both BR biosynthesis and BR signaling

miR156 and *SPL*s likely originated early in plant evolution and are found in all studied land plants, though their exact functions remain unclear [44, 45]. In this study, we found that miR156a and its target *SPL13* regulate lateral branch development in tomato (Fig. 1). miR156 has been reported to regulate lateral branching in several plant species [1, 46–50]. Our previous work has demonstrated that miR156a significantly increases lateral branch number in tomato by downregulating the expression of *SPL13* [1, 8]. The miR156–*SPL13* module also plays important roles in agricultural improvement in several other crops. In rice, the miR156-targeted *OsSPL13* gene is known to control grain size and yield [15]. In maize, miR156–*ZmSPL13* is involved in regulating the vegetative-to-reproductive phase transition [51]. In alfalfa, the miR156–*SPL13* pathway regulates anthocyanin biosynthesis and enhances drought tolerance [52]. In apple, the miR156–*MdSPL13* module plays crucial roles in inducing parthenocarpy and improving salt tolerance [53–56].

The BRC1b transcription factor and DWF have been shown to control lateral branch outgrowth in tomato [29, 38]. In this study, we found that SPL13 could bind the promoters of *DWF* and *BRC1b* (Figs. 3 and 4). We also generated CRISPR-mediated *DWF* knockout (CR-*dwf*) and *BRC1b* OE transgenic lines in the *spl13* mutant background and used them to study the genetic relationships between SPL13 and DWF and BRC1b. Our results showed that the *dwf spl13* double mutant plants and *BRC1b*-OE/flag lines could suppress the increased lateral branch length phenotype of the *spl13* mutant (Figs. 5 and 6). Furthermore, analysis of the contents of BR biosynthesis intermediates in *spl13* mutant plants revealed that SPL13 downregulates BR biosynthesis (Fig. 7B). These findings revealed that both *DWF* and *BRC1b* act downstream of SPL13 in regulation of lateral branch outgrowth in tomato. We propose that SPL13 promotes *BRC1b* expression to inhibit lateral branching and suppresses *DWF* and *BRC1b* expression to promote lateral branching in tomato (Fig. 7C). Given that *DWF* is involved in BR biosynthesis and *BRC1b* functions downstream in the BR signaling pathway, we conclude that the miR156a–*SPL13* module regulates tomato lateral branch outgrowth by influencing both BR biosynthesis and signaling pathways.

### SPL13 regulates branch development by modulating hormone biosynthesis

SLs, a class of carotenoid-derived lactone hormones, play a role in controlling shoot branching, leaf morphology and senescence, anthocyanin accumulation, photomorphogenesis, endosperm development, secondary stem growth, root development, and stress responses [57]. CYP722C, encoding a cytochrome P450 (Solyc02g084930), is a key enzyme that catalyzes the BC-ring closure in SL biosynthesis, leading to the formation of orobanchol, the most prevalent type of the canonical SLs [58]. Our qRT-PCR and EMSA results revealed that *CYP722C* acts downstream of SPL13 (Fig. S1). SPL13 directly inhibits the strigolactone synthesis genes CCD7 and MAX1, thereby regulating lateral branch growth in tomato [36]. These analyses imply that SPL13 may control lateral branch outgrowth in tomato by regulating SL biosynthesis. In addition to regulating SL synthesis and BR synthesis and signaling, SPL13 can also directly repress the transcription of the CK synthesis gene *Isopentenyl transferase 1* (*IPT1*) to regulate lateral branch growth in tomato [36]. These results indicate that SPL13 may regulate hormone-associated signaling pathways and biological processes to influence plant growth and development, particularly lateral branching in tomato. *BRANCHED 1* (*BRC1*) has been reported as an SL-responsive gene in Arabidopsis [59]. In tomato, *BRC1b* is upregulated in the *spl13* mutant due to suppression of the CK biosynthesis gene *IPT1* [36] and DWF regulates bud outgrowth through the BR–*BZR1–BRC1b* pathway [38]. Taken together, we propose that the effects of SLs, BRs, CKs, and the miR156a–*SPL13* module on tomato lateral branch outgrowth are largely mediated through their regulation of *BRC1b* expression.

### Regulation of plant architecture by the miR156a–*SPL13–DWF–BRC1b* genetic pathway

In Arabidopsis, SPL transcription factors influence flowering time, trichome distribution, anthocyanin biosynthesis, and other traits by interacting with relevant gene promoters [21, 23, 60]. miR156a targets the SlSPL13 transcription factor, which directly binds to promoters to regulate target gene expression [8]. Our findings indicate that SPL13 interacts with *DWF* and *BRC1b* promoters, which are associated with lateral branch outgrowth in tomato (Figs 3 and 4).

BRASSINAZOLE-RESISTANT 1 (BZR1) is crucial regulator in BR signaling and a recent study has shown that DWF regulates bud outgrowth through the BR–*BZR1–BRC1b* pathway in tomato [38]. This may help explain our observation in the double mutant line of *spl13/dwf*, where there was lack of regulation of *BRC1b* by SPL13, the lateral branching defects could be attributed mainly to the regulation by the BR–*BZR1–BRC1b* pathway. Our study revealed that *BRC1b* expression levels were notably elevated in *spl13/dwf* plants compared to the *spl13* single mutant (Fig. S2). Based on these data, we hypothesize that DWF is more effective than SPL13 in the regulation of *BRC1b* expression. In other words, the SPL13–*DWF* regulation pathway probably plays a paramount role in the SPL13-mediated regulation of lateral branch outgrowth. These findings suggest a new regulatory pathway involving miR156a, *SPL13*, *DWF*, and *BRC1b* in controlling lateral branch outgrowth in tomato.

Taken together, we propose that SPL13 is crucial in the miR156a–*SPL13–DWF–BRC1b* pathway, regulating lateral branch outgrowth in tomato (Fig. 7C). In this model, SPL13 influences lateral branch development by enhancing *BRC1b* expression. At the same time, SPL13 also represses the expression of *DWF*, through which it regulates lateral branch outgrowth in tomato (Fig. 7C). The miR156a–SPL13 pathway has potential for improving fruit yield and plant architecture in tomato and may be a ubiquitous regulatory pathway in plants.

## Materials and methods

### Plant materials and growth conditions

Cultivar Ailsa Craig (AC) was used as a control in this work and for transformation experiments. WT tomato (AC) and transgenic plants were cultivated in the greenhouse at Huazhong Agriculture University, Wuhan, China (30.4°N, 114.2°E). Recombinant proteins were transiently expressed in *Nicotiana benthamiana*, cultivated at 22°C under a 16-h light and 8-h dark photoperiod.

### Isolation of RNA and analysis of gene expression

Plant total RNA was extracted using Invitrogen’s TRIZOL reagent. Complementary DNAs were synthesized using Toyobo’s M-MLV reverse transcriptase kit. Quantitative PCR (qPCR) analysis was conducted using SYBR Green from Roche. Three biological replicates were analyzed for each genotype. The actin gene BT013524 served as an internal control. Sequences of the primers used in qPCR and other experiments reported in this study are listed in Table S1.

### Genetic constructs and tomato transformation processes

The open reading frames (ORFs) of *DWF* and *BRC1b* were amplified using DNA polymerase (Toyobo). CRISPR-PLANT software was utilized to identify the target sites edited by CRISPR Cas9 technology. The constructs were transformed into AC plants and the *spl13* mutant (which is in the AC genetic background) as described previously [61].

### Transient expression in tobacco leaves

The SPL13 ORF was cloned and amplified into the effector vector pGreen II 62-SK for LUC activity assays. The *BRC1b* and *DWF* promoter fragments were cloned and amplified into the pGreen II 0800-LUC reporter vector [62]. The reporter and effector vectors were transformed into *Agrobacterium tumefaciens* GV2260. We assessed firefly and Renilla luciferase activities using Promega’s dual luciferase assay reagents (USA) 2 days postinfiltration with *Agrobacterium* suspension.

### Electrophoretic mobility shift assays and pull-down assays

Recombinant protein expression plasmids were introduced into BL21 *Escherichia coli* cells following established protocols [8, 63]. The recombinant protein purification and process of EMSA was described as previously [8].

### Chromatin immunoprecipitation

SPL13 was fused with a 6 × FLAG tag (DYKDDDDK) using the pHellsgate 8 vector, followed by immunoprecipitation with anti-FLAG antibodies. Chromatin immunoprecipitation was performed as previously described [8].

### Measurement of brassinolide

For the analysis of endogenous BR levels, 5-week-old young leaves with shoot apices were collected from WT, *DWF*-OX, and *spl13* lines. TY, 6-DCS, BL, and CS (OIChem) were used for preparing corresponding standard solutions. The samples were freeze-dried, and 1 g of lyophilized tissue was ground into a powder in liquid nitrogen and subsequently extracted with 10 ml of ice-cold 95% methanol for 2 h. Following extraction, the homogenate was centrifuged at 10 000 rpm for 5 min at 4°C. The resulting supernatant was purified using columns preloaded with MCX cartridges and eluted with 5 ml methanol. The methanol solution was then dried under nitrogen, dissolved in methanol, filtered through a 0.22-μm membrane. Endogenous brassinosteroids, including TY, 6-DCS, BL, and CS, were quantified using electrospray ionization/high-performance liquid chromatography/tandem mass spectrometry (ESI-HPLC-MS/MS) (ProNets Testing Technology Co.,Ltd, Wuhan, China) following a method described previously [64]. For the ESI-HPLC-MS/MS chromatographic conditions, BL detection was performed on a UPLC (Waters) coupled to a 6500 Qtrap MS equipped with an ESI source (AB SCIEX). Five μl of each sample were injected onto an Agilent Poroshell 120 SB-C18 (2.1 × 150 mm, 2.7 μm) at a constant flow rate of 0.35 ml/min. The mobile phase consisted of water with 0.1% ammonia solution (A) and methanol (B). The gradient program was optimized as follows: 0–1 min, 70% B; 1–14 min, 100% B; 14–14.1 min, 70% B; and 14.1–17 min, 70% B. The column temperature was maintained at 35°C. Mass spectrometry parameters were set for multiple reaction monitoring (MRM) mode. The optimized ESI source parameters were: curtain gas, 30 psi; spray voltage, +5500 V/–4500 V; nebulizer gas, 60 psi; auxiliary gas, 60 psi; and ion source temperature, 400°C. Detailed MRM parameters for each BR were listed in Table S2. System control and data processing were performed using MultiQuant software (Version 3.0.3, SCIEX, USA).

### Statistical analyses

Statistical analysis was performed using SigmaPlot, Excel, and IBM SPSS 22 software. The significant difference was analyzed using Student’s *t*-test. Statistical significance: *P* < 0.05 and *P* < .01.

### Accession numbers

Gene sequences for this study were sourced from the SGN databases with accession numbers: *SlSPL13*, Solyc05g015840; *SlBRC1b*, Solyc06g069240; *SlDWF*, Solyc02g089160 and *SlCYP722C*, Solyc02g084930.

## Supplementary Material

Web_Material_uhag007

## Data Availability

The manuscript and online Supporting Information contain all data underpinning this study’s findings.
