# Supplementary material for: SPL13 controls tomato lateral branch outgrowth by regulating brassinosteroid biosynthesis and signal transduction
Source: Hortic Res. 2026 Apr 10;13(4):uhag007. doi: 10.1093/hr/uhag007 (PMC13095354; doi:10.1093/hr/uhag007)
Supplement: Web_Material_uhag007 [file web_material_uhag007.zip › Supplementary Figure S1-S2.pdf]

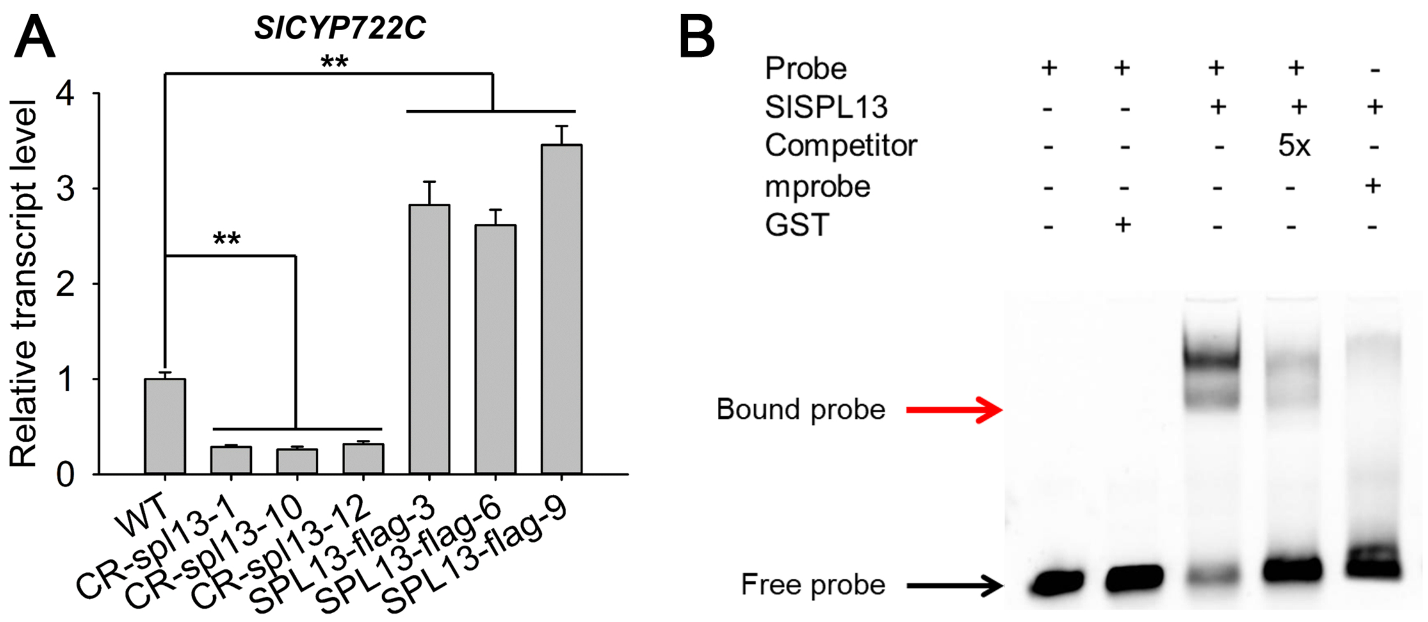

Figure S1. Binding of SPL13 to the promoter of *CYP722C* analyzed by qRT-PCR and electrophoretic mobility shift assay (EMSA). (A) Relative transcript levels of *CYP722C* in the shoot apices of SPL13-FLAG-expressing lines (-3, -6, -9), CR-*spl13* mutant lines (-1, -10, -12) and WT plants. Expression levels were normalized to WT, which was set to 1. Mean values with standard errors (± SE) represent three biological replicates. Asterisks (**) indicate statistically significant differences relative to WT ($P < 0.01$; Student's *t* test). (B) EMSA showing the binding of SPL13 to the promoter region of *CYP722C*. The DNA probe corresponds to the genomic DNA fragment containing the core GTAC motif from *CYP722C* gene promoter. The mutated probe (mProbe) contains the same genomic DNA fragment with the GTAC element replaced by AAGA and served as a negative control. The unlabeled competitor, identical in sequence to the labeled probe, was added at five-fold molar excess (5x). The symbols '-' and '+' denote the absence and presence of the indicated protein or probe. The red arrow indicates the expected position of the DNA-protein complex, and the black arrow denotes the unbound free probe.

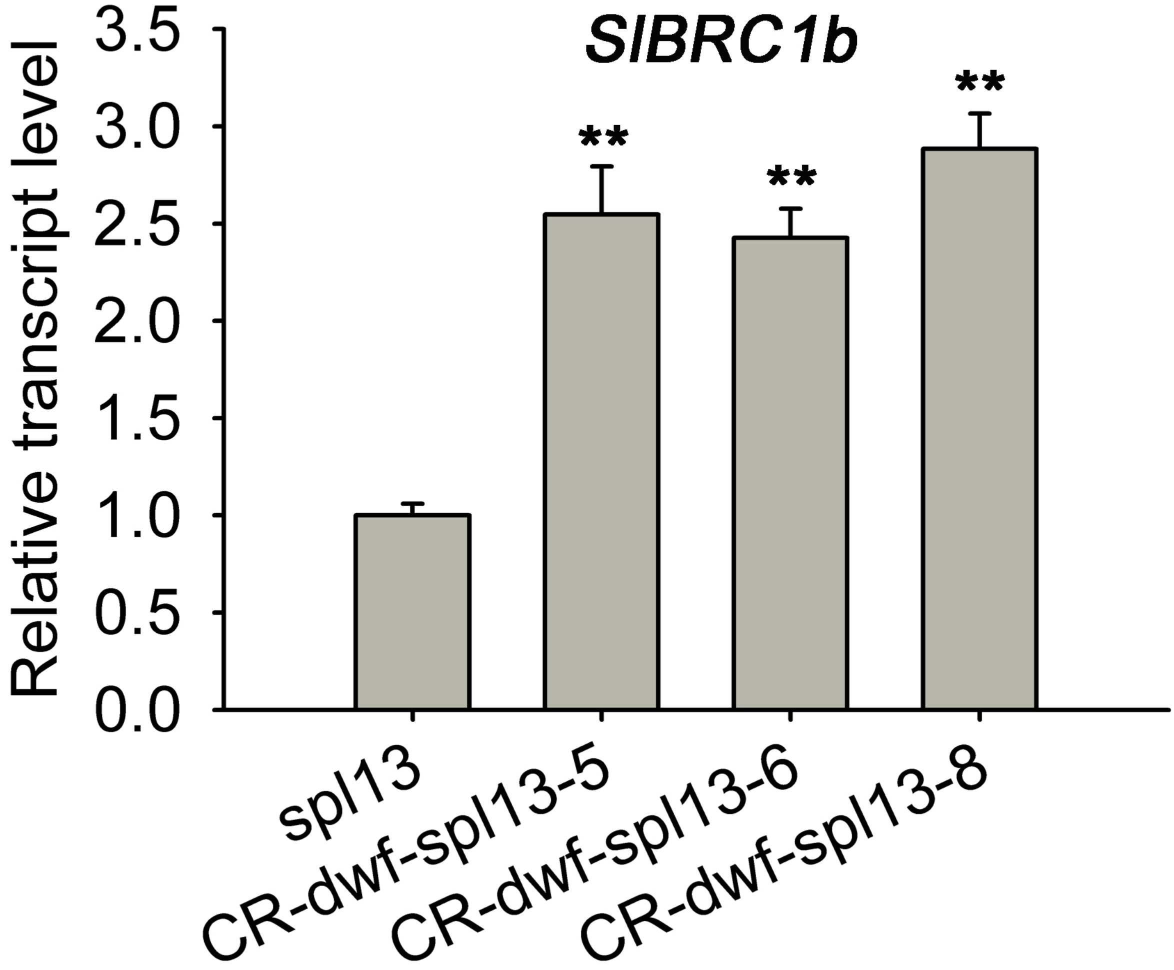

Figure S2. Relative transcript levels of *BRC1b* in shoot apices of *spl13* and *dwf/spl13* plants. Data were normalized to expression in *spl13* plants, which was set to 1. Mean values with standard errors (± SE) are from three replicates. Asterisks (**) indicate statistically significant differences relative to *spl13* ($P < 0.01$; Student's *t* test).
