# Supplementary material for: SPL13 controls tomato lateral branch outgrowth by regulating brassinosteroid biosynthesis and signal transduction
Source: Hortic Res. 2026 Apr 10;13(4):uhag007. doi: 10.1093/hr/uhag007 (PMC13095354; doi:10.1093/hr/uhag007)
Supplement: Web_Material_uhag007 [file web_material_uhag007.zip › Supplementary Table S1-S2.pdf]

Table S1 Primer sequences utilized in this study.

Primer name	Sequence (5'-3')
PBRC1b-1-PAbAi-FW	AGCTTGAATTCGAGCTCGGTACCCCAATATCGCTAAAGGCTTTAGAGA
PBRC1b-1-PAbAi-RV	ACATACAGAGCACATGCCTCGAGGTCACCGGTCAAACTATATTGACC
PBRC1b-2-PAbAi-FW	AGCTTGAATTCGAGCTCGGTACCTAGGTTTCATTCATTGAATTGATGG
PBRC1b-2-PAbAi-RV	ACATACAGAGCACATGCCTCGAGCCAACAAATTGAAGGATGTTTCC
PBRC1b-3-PAbAi-FW	AGCTTGAATTCGAGCTCGGTACCAATTGGCAAATAGTGTTTGTCCA
PBRC1b-3-PAbAi-RV	ACATACAGAGCACATGCCTCGAGTGACTTTGTCATCCTGAGCCG
PBRC1b-4-PAbAi-FW	AGCTTGAATTCGAGCTCGGTACCGGATGGGTCTAGTTAGGGTTGTG
PBRC1b-4-PAbAi-RV	ACATACAGAGCACATGCCTCGAGGAGGATACATTTGTGGAGTAGCTGT
PDWF-1-PAbAi-FW	AGCTTGAATTCGAGCTCGGTACCCATAAAATACACAAAGTGCAATGCC
PDWF-1-PAbAi-RV	ACATACAGAGCACATGCCTCGAGCATTTAAGGATGTTCGATGTATTGC
PDWF-2-PAbAi-FW	AGCTTGAATTCGAGCTCGGTACCGAGATGAGATATTATTATCAAAATCGTCA
PDWF-2-PAbAi-RV	ACATACAGAGCACATGCCTCGAGGGTTACCATGTGAATATAAGTGGACAC
PDWF-3-PAbAi-FW	AGCTTGAATTCGAGCTCGGTACCCTTCTAATAGGGTGGTGTGCATGT
PDWF-3-PAbAi-RV	ACATACAGAGCACATGCCTCGAGGGCTTATTTTGCCAAAAATCTAATG
DWF-0800-FW	CACTATAGGGCGAATTGGGTACCATCCTAATGAGTAATCAGGCGACC
DWF-0800-RV	TATGTTTTTGGCGTCTTCCATGGTGATGCACCTCAAAATTTCAAAAG
DWF-DT1-FW	GAATCTAACAGTGTAGTTTGGGCCAACCCATAGTACCAGGGTTTTAGAGCTAGAAATAG
DWF-DT2-RV	GCTATTTCTAGCTCTAAAACGGCTCTTTGGTTTTTCATGACAAACTACACTGTTAGATT
DWF-DET-FW	CCACAAGGTTAGTGGTCCCTGT
DWF-DET-RV	TATCATAGACTGTGGGTATCCTGGG
SPL13-GST-FW	TTCCGCGTGGATCCCCGGAATTCATGGAATCATCATCATCGTCATC
SPL13-GST-RV	CAGTCACGATGCGGCCGCTCGAGTTAGTCCCACATAAAGGCTAGTGTT
β -actin-Fw	ATGGCAGACGGAGAGGATATTCA
β -actin-Rv	GCCTTTGCAATCCACATCTGCTG
BRC1b-OE/flag-FW	GATGACGATGACAAGGAATTCATGTATCCTCCAAGCAACAATAACT
BRC1b-OE/flag-RV	GTCCTTGTAATCCATGAATTCGCCTTCCCATAGCTTTCCACA
BRC1b-0800-FW	CACTATAGGGCGAATTGGGTACCGCCATTATTCTCCTCTCCTTTACAG
BRC1b-0800-RV	TATGTTTTTGGCGTCTTCCATGGTCCTTGTAGTTCGTGGTTGCAA
BRC1b-Probe-P2.5Kb-FW	GAGATTCAAGGCCAGAAAGGTACCATGCAAGCTGTTATT
BRC1b-probe-P2.5Kb-RV	AATAACAGCTTGCATGGTACCTTTCTGGCCTTGAATCTC
BRC1b-mProbe-P2.5Kb-FW	GAGATTCAAGGCCAGAAAGAAGACATGCAAGCTGTTATT
BRC1b-mProbe-P2.5Kb-RV	AATAACAGCTTGCATGTCTTCTTTCTGGCCTTGAATCTC
BRC1b-Probe-PTSS-FW	GTCAAATTACTGTATACAGTACATTGAACTAGCTAGGGG
BRC1b-probe-PTSS-RV	CCCCTAGCTAGTTCAATGTACTGTATACAGTAATTTGAC
BRC1b-mProbe-PTSS-FW	GTCAAATTACTGTATACAAAGAATTGAACTAGCTAGGGG

BRC1b-mProbe-PTSS-RV	CCCCTAGCTAGTTCAATTCTTTGTATACAGTAATTTGAC
BRC1b-ChIP-CK-FW	CACATTAAATTTAGGGATGTTTTCG
BRC1b-ChIP-CK-RV	TGCTCGTATAGAACTTGGGATCAC
BRC1b-ChIP-TTS-FW	TCATCACTTTGGTCAATCCATACAG
BRC1b-ChIP-TTS-RV	TTCCCCTAGCTAGTTCAATGTACTGT
BRC1b-ChIP-P2.5Kb-FW	GCACGTGTAGTTTCAAACGCAG
BRC1b-ChIP-P2.5Kb-RV	GCTTGCATGGTACCTTTCTGGC
BRC1b-Q-FW	TGTATCCTCCAAGCAACAATAACTG
BRC1b-Q-RV	CATCATCTCCTTTCTTTTCGCC
CYP722C-Probe-FW	CCATGCCGGCTTACCAAGGTACTCACCACATTCGCTAAAA
CYP722C-probe-RV	TTTTAGCGAATGTGGTGAGTACCTTGGTAAGCCGGCATGG
CYP722C-mProbe-FW	CCATGCCGGCTTACCAAGAAGATCACCACATTCGCTAAAA
CYP722C-mProbe-RV	TTTTAGCGAATGTGGTGATCTTCTTGGTAAGCCGGCATGG
CYP722C-Q-FW	GTGTTTGTTCCAAGTGTTGAGGG
CYP722C-Q-RV	CGCCTAATCCTTTTGTGACTCTC
DWF-Probe-FW	CATAGATTTTGGGTTTGAGTACTGAGTCATACCCCAAAT
DWF-probe-RV	ATTTGGGGTATGACTCAGTACTCAAACCCAAAATCTATG
DWF-mProbe-FW	CATAGATTTTGGGTTTGAAAGATGAGTCATACCCCAAAT
DWF-mProbe-RV	ATTTGGGGTATGACTCATCTTTCAAACCCAAAATCTATG
DWF-Q-FW	CTCTGTCTGTGAGGTGGTTTGGT
DWF-Q-RV	CCATAGTACCAGGGGGCAAGT
DWF-ChIP-CK-FW	GAAAGAGAATTTACCCTCCAATGTT
DWF-ChIP-CK-RV	TGACTCTTCAACTAAAATCTCCCAC
DWF-ChIP-TSS-FW	GATTGCATGAAATACAAGTTGCACT
DWF-ChIP-TSS-RV	TGCTTCAAAATCTTATATTTGGGGT

Table S2. Mass spectrometry parameters of BL measurement.

Substance	Polarity	Precursor ion (m/z)	Product ion (m/z)	Declustering potential (V)	Collision energy (V)
BL	+	481.2	445.3/315.3*	42	13/19
CS	+	465.3	429.4*/269.1	44	21/27
6-DCS	-	449.4	128.9/377.2*	-60	-31/-29
TY	+	449.5	431.3/175.0*	95	17/40

Note: The ions marked with * are quantitative ions.
